# Carnitine Supplementation Attenuates Sunitinib-Induced Inhibition of AMP-Activated Protein Kinase Downstream Signals in Cardiac Tissues

**DOI:** 10.1007/s12012-018-9500-0

**Published:** 2019-01-14

**Authors:** Mohamed M. Sayed-Ahmed, Badr I. Alrufaiq, Ammar Alrikabi, Mashan L. Abdullah, Mohamed M. Hafez, Othman A. Al-Shabanah

**Affiliations:** 1 Pharmacology, Cancer Biology Department, National Cancer Institute, Cairo University, Cairo, 11796 Egypt; 2 Department of Pharmacology and Toxicology, College of Pharmacy, King Saud University, P.O. Box 2457, Riyadh, 11451 Kingdom of Saudi Arabia; 3 Pathology Department, College of Medicine, King Saud University, P.O. Box 2457, Riyadh, 11451 Kingdom of Saudi Arabia; 4 King Abdullah International Medical Research Center, P.O. Box 2457, Riyadh, 11451 Kingdom of Saudi Arabia; 5 Virology and Immunology Units, Cancer Biology Department, National Cancer Institute, Cairo University, Cairo, 11796 Egypt

**Keywords:** Sunitinib, CPT I, Cardiotoxicity, l-carnitine

## Abstract

This study has been initiated to investigate whether sunitinib (SUN) alters the expression of key genes engaged in mitochondrial transport and oxidation of long chain fatty acids (LCFA), and if so, whether these alterations should be viewed as a mechanism of SUN-induced cardiotoxicity, and to explore the molecular mechanisms whereby carnitine supplementation could attenuate SUN-induced cardiotoxicity. Adult male Wister albino rats were assigned to one of the four treatment groups: Rats in group 1 received no treatment but free access to tap water for 28 days. Rats in group 2 received l-carnitine (200 mg/kg/day) in drinking water for 28 days. Rats in group 3 received SUN (25 mg/kg/day) in drinking water for 28 days. Rats in group 4 received the same doses of l-carnitine and SUN in drinking water for 28 days. Treatment with SUN significantly increased heart weight, cardiac index, and cardiotoxicity enzymatic indices, as well as severe histopathological changes. Moreover, SUN significantly decreased level of adenosine monophosphate-activated protein kinase (AMPKα2), total carnitine, adenosine triphosphate (ATP) and carnitine palmitoyltransferase I (CPT I) expression and significantly increased acetyl-CoA carboxylase-2 (ACC2) expression and malonyl-CoA level in cardiac tissues. Interestingly, carnitine supplementation resulted in a complete reversal of all the biochemical, gene expression and histopathological changes-induced by SUN to the control values. In conclusion, data from this study suggest that SUN inhibits AMPK downstream signaling with the consequent inhibition of mitochondrial transport of LCFA and energy production in cardiac tissues. Carnitine supplementation attenuates SUN-induced cardiotoxicity.

## Introduction

Sunitinib (SUN) is an oral multitargeted tyrosine kinase inhibitor which has been approved in the treatment of gastrointestinal stromal tumors and metastatic renal cell carcinoma [1]. Although SUN has proved efficacy and increased the survival of patients with cancer, its optimal clinical usefulness is usually limited secondary to the development of cardiotoxicity [2–5]. Cardiac events arising from SUN include congestive heart failure, reduction in left ventricular ejection fraction, hypertension, myocardial infarction, and thromboembolism [6, 7]. Histopathological examination of endomyocardial biopsies from SUN-treated patients who developed heart failure revealed cardiomyocyte hypertrophy and mitochondrial abnormalities including swelling, membrane whorls, and effaced cristae [4, 6]. Several in vitro and in vivo experimental studies reported that SUN induces its cardiotoxicity by several mechanisms [8–13]. Earlier in vitro and in vivo studies have demonstrated that SUN induces its cardiotoxicity by inhibition of adenosine monophosphate-activated protein kinase (AMPK) [14–16]. Using cardiomyocytes and SUN-induced cardiomyopathic mouse models, Kerkela et al. confirmed that SUN decreased expression and activity of AMPK which significantly decreased phosphorylation of its substrate, acetyl-CoA carboxylase (ACC) in cardiac tissues [17]. Cardiac AMPK complexes consist predominantly of alpha 2, beta 2, and gamma 2 subunits [18] and act as an important regulator of LCFA oxidation and glucose uptake in the heart [19]. In cardiac tissues, activation of AMPK leads to phosphorylation and inhibition of ACC2, with the consequent increase in the transport LCFA from cytoplasmic compartment into mitochondria, where β-oxidation enzymes are located for energy production [20]. Conversely, inhibition of AMPK inhibits phosphorylation and activates ACC2 leading to inhibition of mitochondrial transport and oxidation of LCFA [21].

l-carnitine is an obligatory cofactor for mitochondrial transport and oxidation of LCFA which supply the heart with approximately 60–90% of ATP [22, 23]. In the heart, l-carnitine plays several vital metabolic functions via increasing intramitochondrial CoA-SH/Acetyl-CoA ratio [24], regulation of apoptosis and inflammation, protection from oxidative stress and modulation of cellular stress response and gene expression [25–28]. Earlier studies in our laboratory have demonstrated that carnitine supplementation attenuates cardiotoxicity induced by doxorubicin [29], cyclophosphamide and ifosfamide [30] via modulating the expression of key genes involved in mitochondrial transport and oxidation of LCFA. Although several studies have documented that SUN induces its cardiotoxicity by inhibiting AMPK and altering energy status in mitochondria, the effects of SUN on AMPK downstream signal and the role of substrate utilization are not studied to date. Accordingly, the current study has been initiated to investigate whether SUN alters the expression of key genes engaged in mitochondrial transport and oxidation of LCFA, and if so, whether these alterations should be viewed as a mechanism during development of SUN-induced cardiotoxicity. Other important goal of this study is to further explore the molecular mechanisms whereby carnitine supplementation could alter SUN-induced cardiotoxicity.

## Materials and Methods

### Materials

Sunitinib has been purchased from LC Laboratories (Woburn, MA, USA). It has been supplied in non-commercial glass brownish bottle containing 10-gm yellow powder and was freshly dissolved in drinking water prior to administration. l-carnitine has been graciously given by Dr. Zaven Orfalian, Sigma-Tau Pharmaceuticals, Pomezia, Italy. It has been supplied as white powder in non-commercial plastic bottles contains 100 g and it was freshly dissolved in drinking water prior to administration. Primers and probes were designed using Primer Express 3.0 (Applied biosystem, life technology, USA) and purchased from Metabion International AG (Germany). Diagnostic kits for measurement of CK-MB and LDH were obtained from Greiner Diagnostic (GmbH, Germany). Carnitine assay kits were obtained from Biosentec (Hall Gilbert Durand, France). ACC2 assay kit was purchased from MyBiosource, Cat No. MBS7239707. AMPK α2 ELISA kit was purchased from (LifeSpan BioSciences, Inc, Cat No. LS-F21864). Carnitine palmitoyltransferase (CPTI) assay kit was purchased from (MyBiosource, Cat No. MBS2602676). Malonyl-CoA decarboxylase (MCD) assay kit was purchased from (MyBiosource, Cat No. MBS2601663). All other chemicals used were of the highest analytical grade.

### Animals

Adult male Wistar albino rats, weighing 180–200 g, were obtained from the Animal Care Center, College of Pharmacy, King Saud University, Riyadh, Kingdom of Saudi Arabia and were housed in metabolic cages under controlled environmental conditions (25 °C and a 12-h light/dark cycle). Animals had free access to pulverized standard rat pellet food and tap water. The protocol of this study has been approved by Research Ethics Committee of College of Pharmacy (E.A.C.C-11/2016), King Saud University, Riyadh, Kingdom of Saudi Arabia.

### Experimental Design

A total of 40 adult male Wistar albino rats were used and divided at random into 4 groups of 10 animals each. Rats of group 1 (control group) received no treatment but free access to tap water for 28 days. Rats of group 2 (SUN-treated group) received SUN (25 mg/kg /day) in drinking water for 28 days. Rats in group 3 (carnitine-supplemented group) received l-carnitine (200 mg/kg/day) in drinking water for 28 days. Rats in group 4 (SUN plus l-carnitine group) received l-carnitine (200 mg/kg/day) and SUN (25 mg/kg/day) in drinking water for 28 days. To ensure correct dosage of the treatment protocol, the concentrations of SUN and l-carnitine in drinking water were adjusted according to daily water intake. In the current study, selected doses and route of administration of both SUN and l-carnitine were adopted from previous studies in rodents [9, 13, 29–31]. The dose of l-carnitine (200 mg/kg/day) selected in our study is clinically relevant for human use since the average daily dose of l-carnitine in cancer patients and in the treatment of cancer chemotherapy-induced organs toxicity is up to 4 g/day with no toxicity [32, 33]. The human equivalent dose (HED) for l-carnitine (200 mg/kg/day) equals 2.27 g is relevant for human use and has been calculated according to Nair and Jacob [34] as the following: HED (mg/kg) = Animal dose (mg/kg) × (Animal Km ÷ Human Km). HED (mg/kg) = Rat (200 mg/kg) × (6/37), HED (mg/kg) = 200 × 0.162 = 32.43 mg/kg, HED for 70 kg patient = 32.43 × 70 = 2.27 g. Therefore, 200 mg/kg of l-carnitine in rat selected in our study is equivalent to 2.27 g human of 70 kg. Twenty-four hours after the end of the treatment protocol, animals were sacrificed by decapitation after exposure to ether in a desiccator kept in a well-functioning hood and blood samples were obtained. Serum was separated for determination of cardiotoxicity enzymatic indices (LDH and CK-MB), AMPKα_2_ and total carnitine. Immediately after withdrawal of blood samples, hearts were then quickly isolated and removed intact, washed with ice-cold 0.9% saline solution and weighed. Cardiac index (CI) was calculated as the heart weight to the body weight ratio (g/100 g) in each animal. Then, isolated hearts were divided into two parts. The first part from each heart was used for determination of total carnitine, AMPKα2, ATP, malony-CoA, CPT I, MCD and ACC2 levels, whereas the second part was used for mRNA expression of ACC2, CPT I, MCD and histopathological studies.

### Methods

#### Quantification of mRNA Expression by Real-Time Polymerase Chain Reaction

Total RNA was extracted from heart tissues using Trizol method as previously described [35]. In brief, RNA was extracted by homogenization of cardiac tissues in TRIzol reagent (GibcoBRL) at maximum speed for 90–120 s. The homogenate was incubated for 5 min at room temperature. A 1:5 volume of chloroform was added, and the tube was vortexed and subjected to centrifugation at 12,000×*g* for 15 min. The aqueous phase was isolated, and the total RNA was precipitated by cold absolute ethanol. After centrifugation and washing, the total RNA was finally eluted in 20 µl of diethyl pyrocarbonate-treated water. The quantity was characterized using a UV spectrophotometer (NanoDrop 8000, Thermo Scientific, USA). The isolated RNA has a 260/280 ratio of 1.9–2.1. First-strand cDNA was synthesized from 1 µg of total RNA by reverse transcription with a SuperScript™ first-strand synthesis system kit (Invitrogen, CA, USA), according to the manufacturer’s instructions. Real-time reaction was performed using the KAPA PROBE FAST qPCR kit master mix (KAPA Biosystems, USA) and the 2−ΔΔCt method. GAPDH gene was used as the endogenous control. PCR assay was optimized by varying the PCR conditions such as the concentration of cDNA, primers and probes, amplification cycle number and annealing temperature. Briefly, a standard 25 µl reaction mixture contained in final concentration of 1 × KAPA PROBE FAST qPCR master mix buffer, 0.4 µM of each forward and reverse primers, 0.2 µM Probe for H-FABP, CPT IB, ACC2, MCD and GAPDH (Table 1), 100 ng of cDNA and RNase, DNase free water. The reaction was done in an ABI 96-Well optical reaction plate placed on ice before cDNA template was added. The standard thermal cycling conditions of initial 50 °C for 2 min and 95 °C for 10 min followed by 40 cycles at 95 °C for 15 s and 60 °C for 1 min were used. All reactions were performed using an ABI 7500 System (Applied Biosystem, USA). Experiments were performed in triplets for all data points. Each qPCR reaction included no-template controls.

**Table 1:** Primers and probe sequence of the GAPDH, CPT IB, ACC 2 and MCD genes

Gene name	Forward primer	Reverse primer	Probe
GAPDH	5′-TGGCCTCCAAGGAGTAAGAAAC-′3	5′-GGCCTCTCTCTTGCTCTCAGTATC-′3	FAM-CTGGACCACCCAGCCCAGCAA-TAMRA
CPTIB	5′-CAAACATCACTGCCCAAGCTT-′3	5′-GGCCGCACAGAATCCAAGT-′3	FAM-TGTGCCAGCCACAATTCACCGG-TAMRA
ACC2	5′-CTTTTCTAGGTCCCCGAGTGA-′3	5′-CTTCCGCTCCAGGGTAGAGTT-′3	FAM-AGGCTCTCCTCCACCATTGTAGCCCA-TAMRA
MCD	5′-CAGAGGACCGGCTACGCTAT-′3	5′-CAGCTTACTGATGTGGTGGAAGAG-′3	FAM-CCCTCGTGCCGCGATACCGT-TAMRA

#### Determination of Malonyl-CoA and Adenosine Triphosphate Levels in Rat Cardiac Tissue Using HPLC

Malonyl-CoA and adenosine triphosphate levels were determined in heart tissues using HPLC system (Jasco Corporation, Ishikawa-Cho, Hachioji, Tokyo, Japan) According to Lysiak et al. [36] and Botker et al. [37], respectively. In brief, heart tissue was homogenized in ice-cold 6% perchloric acid, centrifuged at 1000 rpm for 15 min at 0.5 °C, and the supernatant fluid was injected into HPLC after neutralization to pH 6–7. Chromatographic separation was performed using ODS-Hypersil, 150 × 4.6 mm I.D., 5 µm column (Supelco SA, Gland, Switzerland). For malonyl-CoA detection, the UV detector was operated at 254 nm and set at 0.005. The gradient elution was performed using two mobile phases including, 220 mM potassium phosphate containing 0.05% dithioglycol (A) and 98% methanol, 2% chloroform (B). The flow rate was 0.6 ml/min and the gradient was as follows: at zero time, 94% A and 6% B; at 8 min, 92% A and 8% B; at 14 min, 87% A and 13% B; at 25 min, 80% A and 20% B; at 40 min, 55% A and 45% B; at 45 min, 55% A and 45% B; and at 60 min, 94% A and 6% B. For ATP detection, the Isocratic elution was performed at a flow rate of 1.2 ml/min, using 75 mM ammonium dihydrogen phosphate as mobile phase. The ATP peaks were eluted at 3.2 min and the UV detector was operated at 254 nm.

#### Determination of Total Carnitine in Serum and Cardiac Tissues

Total carnitine concentration was determined in serum and cardiac tissues according to the method reported by Prieto et al. [38]. In brief, carnitine reacts with acetyl-CoA forming acetylcarnitine in a reaction mediated by carnitine acetyltransferase enzyme. The liberated CoA-SH reacts with 5,5-dithiobis-(2-nitrobenzoic acid) and forming thiophenolate ion, whose generation is proportional to the amount of carnitine and can be measured spectrophotometrically at 412 nm.

#### Determination of AMPKα2, ACC2, CPTI, and MCD Levels in Cardiac Tissues

The levels of AMPKα2, ACC2, CPTI, and MCD were quantitatively determined in cardiac tissue homogenate using ELISA kits. Samples from cardiac tissue homogenates were prepared by subjecting tissue homogenates to three freeze (− 20 °C)/thaw (room temperature) cycles to further break the cell membranes. After that, the homogenates were centrifugated for 15 min at 5000 rpm and the levels of AMPKα2, ACC2, CPTI, and MCD were measured in the supernatant immediately according to manufacturer’s instructions.

#### Assessment of Serum Creatine Kinase (CK-MB) and Lactate Dehydrogenase (LDH) Activity

Serum activities of LDH and CK-MB were determined according to the methods of Buhl and Jackson [39] and Wu and Bowers [40], respectively.

#### Histopathological Examination of Cardiac Tissues

Heart specimens from each group were removed to be examined histopathologically. They were fixed in 10% neutral buffered formalin for 24 h until the tissue became hard enough to be sectioned. Tissues were then embedded in paraffin wax, sectioned at 3 µm and stained with hematoxylin for 10 min then counterstained in eosin for 1 min followed by rapid rinsing in distilled water. To detect the presence of myocardial fibrosis, heart sections were stained with Masson Trichrome (MT) connective tissue staining. Finally, sections were dehydrated and examined using a light microscope and photographed.

#### Statistical Analysis

Differences between obtained values (mean ± SEM, *n* = 10) were carried out by one-way analysis of variance (ANOVA) followed by the Tukey–Kramer multiple comparison test. *p* ≤ 0.05 was taken as a criterion for a statistically significant difference.

## Results

**Fig. 1:**
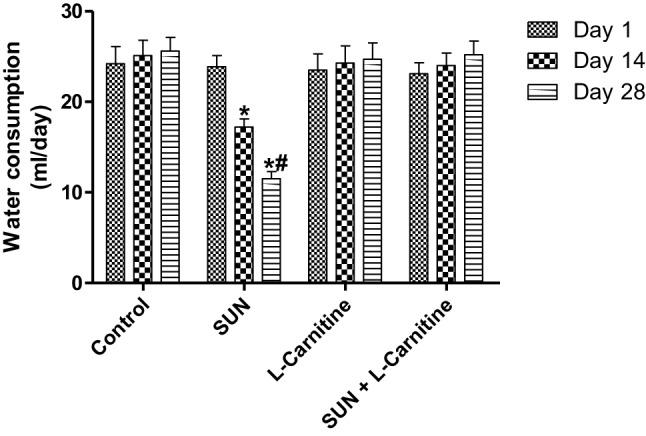
Effects of Sunitinib, l-carnitine, and their combination on water consumption of rats. Data are presented as mean ± S.E.M. (*n* = 10). Asterisk and ash symbols indicate significant change from day 1 and day 14, respectively, at *p* < 0.05 using ANOVA followed by Tukey–Kramer as a post ANOVA test

To ensure correct dosage of the treatment protocol, the concentrations of SUN and l-carnitine in drinking water were adjusted according to water intake. In this regard, rats were individually housed in separate cages for calculating the daily water consumption. As shown in Fig. 1, no significant change in daily water consumption in control and carnitine-treated rats was observed. However, in SUN-treated rats, the daily water consumption was significantly decreased by 28% and 52% in day 14 and day 28, respectively, as compared to day 1 of the treatment protocol. Interestingly, administration of l-carnitine concomitant with SUN resulted in a complete reversal of the SUN-induced decrease in water consumption to the control values.

**Fig. 2:**
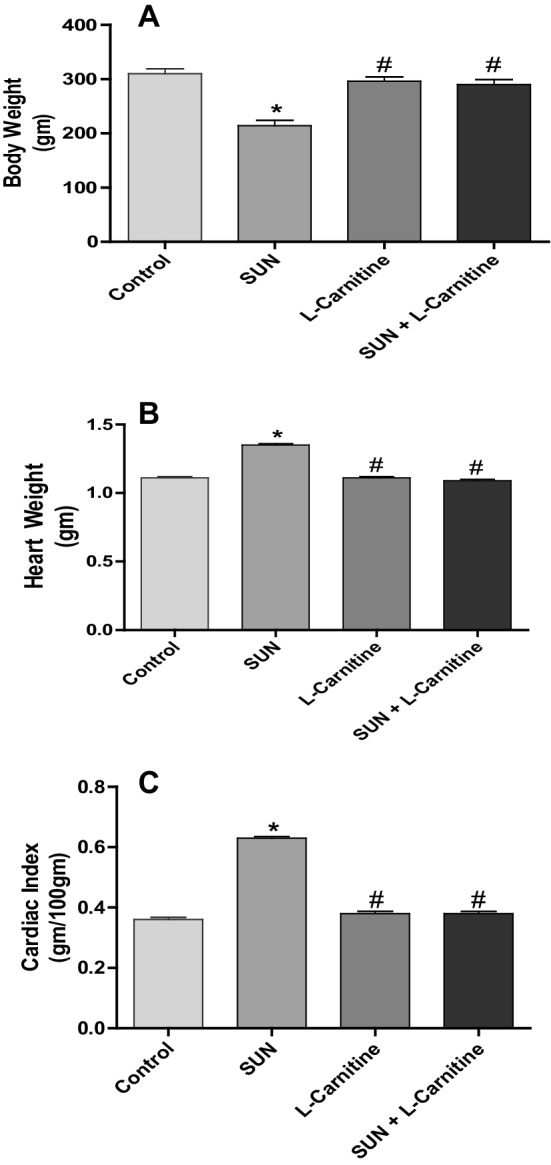
Effects of Sunitinib, l-carnitine and their combination on **a** body weight, **b** heart weight and **c** cardiac index of rats. Data are presented as mean ± S.E.M. (*n* = 10). Asterisk and ash symbols indicate significant change from control and SUN, respectively, at *p* < 0.05 using ANOVA followed by Tukey–Kramer as a post ANOVA test

Figure 2 shows the effect of SUN, l-carnitine and their combination on body weight (A), heart weight (B) and cardiac index (C) in rats. Daily administration of SUN (25 mg /kg) for 28 successive days resulted in a significant 31% decrease in body weight (A) and a significant 23% and 75% increase heart weight (B) and cardiac index (C), respectively, as compared to the control group. On the other hand, carnitine supplementation (200 mg/kg/day) for 28 successive days showed non-significant changes. Interestingly, daily administration of l-carnitine concomitant with SUN resulted in a complete reversal of SUN-induced decrease in body weight and increase in heart weight and cardiac index to the control values.

**Fig. 3:**
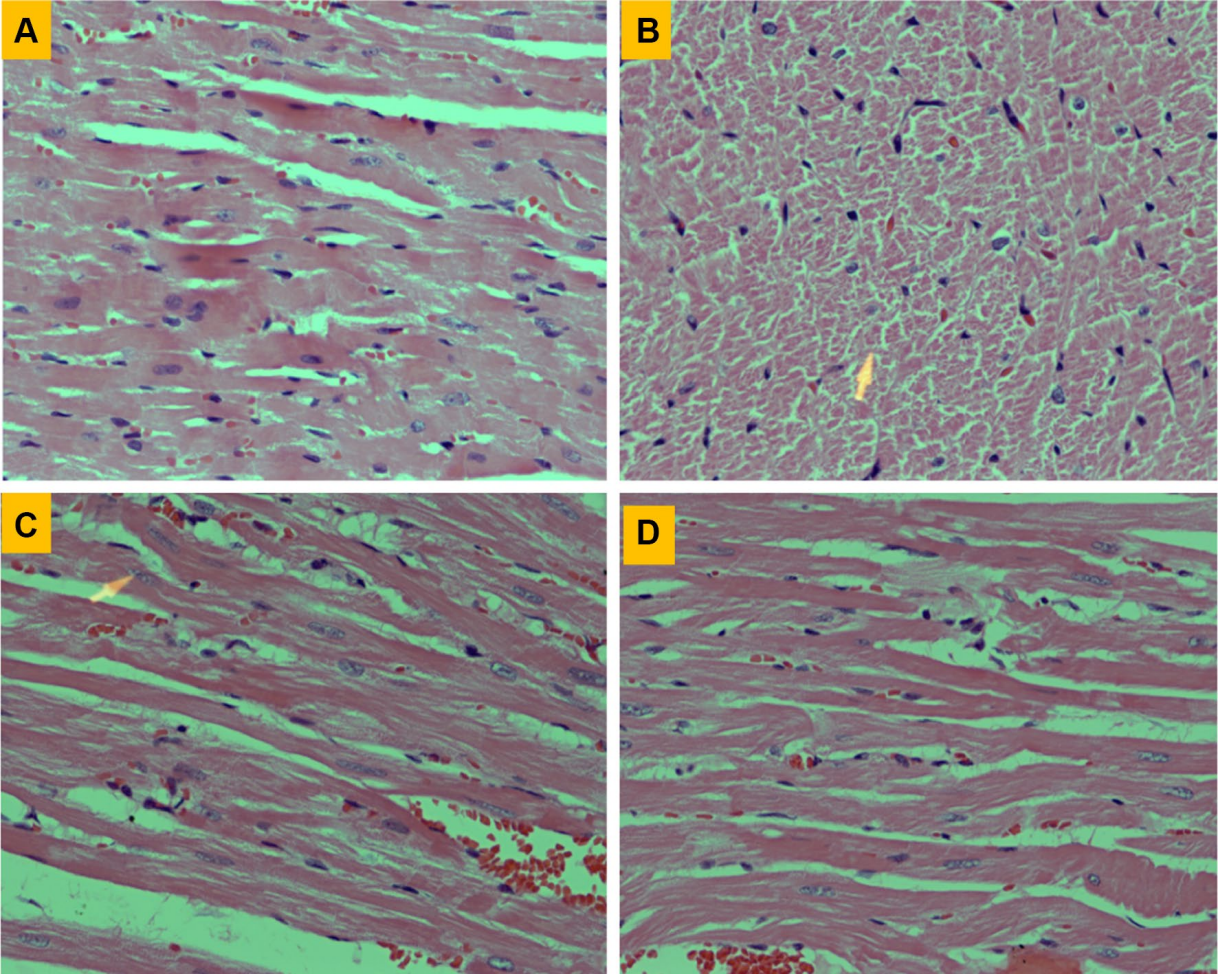
Effects of Sunitinib, l-carnitine and their combination on histopathological changes in cardiac tissues stained with H & E stain. **a** Heart from control rat showing normal muscle fibers (× 400). **b** Heart of rat treated with SUN alone showing scattered chronic inflammatory cells with focal fragmentation of myocardial fibers and loss of nuclei, arrowhead (× 400). **c** Heart of rat treated with l-carnitine alone showing almost completely normal myocardial fibers (× 400). **d** Heart of rats treated with SUN plus l-carnitine showing marked reduction in the amount of connective tissue fibers indicative of effective protection against myocardial fibrosis (× 400)

Figure 3 shows the histopathological changes in cardiac tissues induced by SUN in normal and carnitine-supplemented rats. Section of myocardium from control rats showed bundles of normal muscle fibers and no significant pathological changes (Fig. 3a). On the other hand, section of myocardium treated daily with SUN (25 mg/kg) for 28 successive days (Fig. 3b) showed severe histopathological changes which are consistent with cardiotoxicity manifested as scattered chronic inflammatory cells with focal fragmentation of myocardial fibers and loss of nuclei (arrowhead). Section of myocardium treated with l-carnitine alone (200 mg/kg/day) for 28 successive days showed almost completely normal myocardial fibers (Fig. 3c). Interestingly, heart specimens from rats treated with SUN plus l-carnitine showed normal myocardial fibers with residual degenerate fibers in the upper parts of the picture (Fig. 3d) indicating good protection in comparison to SUN alone (Fig. 3b).

**Fig. 4:**
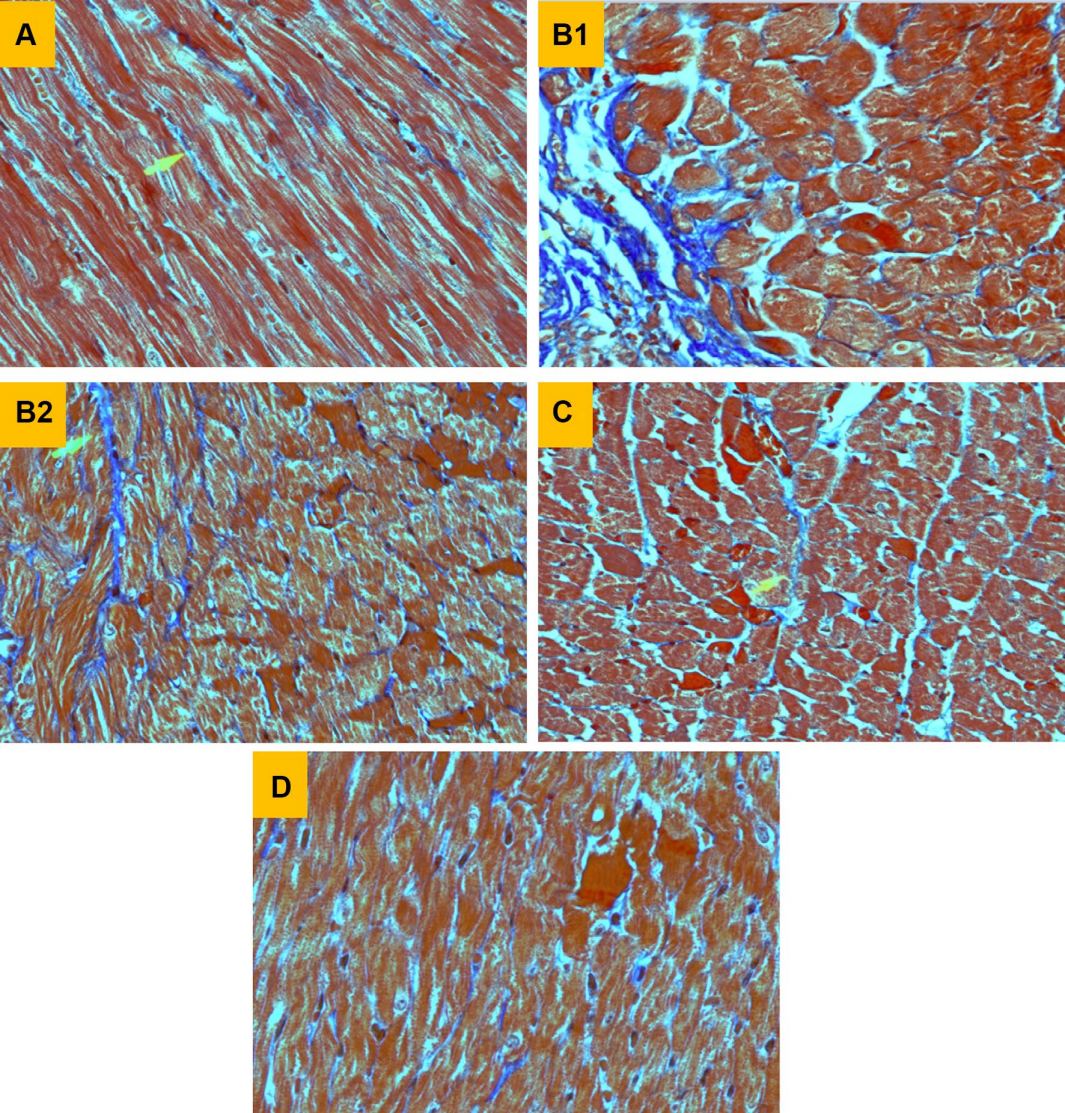
Effects of Sunitinib, L-carnitine and their combination on histopathological changes in cardiac tissues stained with Masson Trichrome (MT) connective tissue stain. **a** Heart from control rat showing normal myocardial fibers (× 400). **b** Heart of rat treated with SUN alone showing marked fibrosis (blue colored fibers) with extension to interstitial tissue between myofibers (B1, × 600) and interstitial fibrosis characterized by the presence of many “blue colored” connective tissue fibers, arrowhead, (B2, × 600). **c** Heart of rat treated with l-carnitine alone showing normal myocardial fibers with only thin blue-colored collagen fibers, arrowhead, (× 400). **d** Heart of rat treated with SUN plus l-carnitine showing marked reduction in the amount of connective tissue fibers (× 400)

To detect the presence of SUN-induced myocardial fibrosis, heart specimens were stained with Masson Trichrome (MT) connective tissue stain (Fig. 4). Heart specimens from control rats stained with MT showed normal myocardial fibers with very scanty blue-colored collagen fibers (Fig. 4a). Section of myocardium treated with SUN alone for 28 successive days showed marked fibrosis (blue-colored fibers) with extension to interstitial tissue between myofibers (Fig. 4b1) and interstitial fibrosis characterized by the presence of many “blue colored” connective tissue fibers, arrowhead, (Fig. 4b2). On the other hand, section of arrowhead (Fig. 4c) treated with l-carnitine alone for 28 successive days showed normal myocardial fibers with only thin blue-colored collagen fibers. Interestingly, heart specimens from rats treated with SUN plus l-carnitine showed marked reduction in the amount of connective tissue fibers indicative of effective protection against myocardial fibrosis (Fig. 4d).

**Fig. 5:**
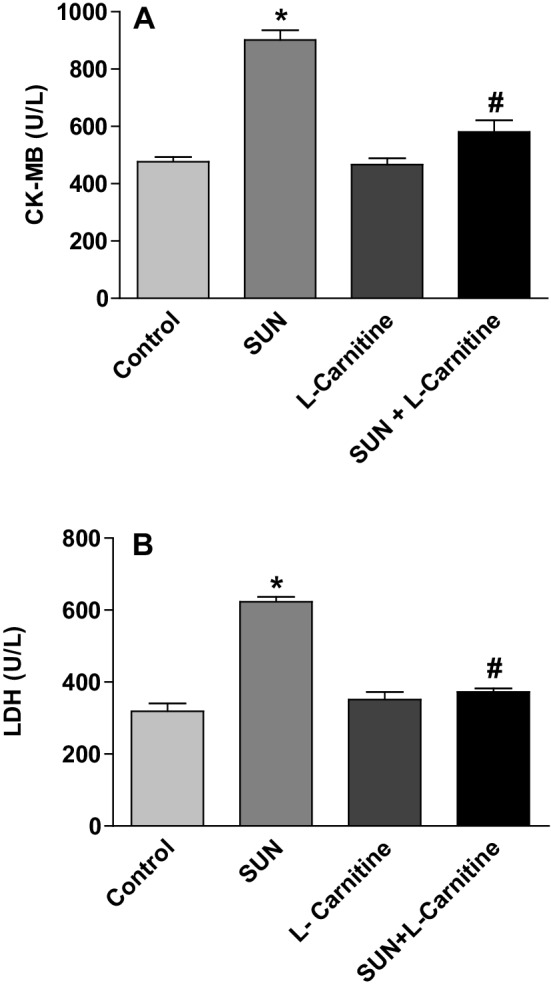
Effects of Sunitinib, l-carnitine, and their combination on serum cardiotoxicity enzymatic indices, CK-MB (**a**) and LDH (**b**), in rats. Data are presented as mean ± S.E.M. (*n* = 10). Asterisk and ash symbols indicate significant change from control and SUN, respectively, at *p* < 0.05 using ANOVA followed by Tukey–Kramer as a post ANOVA test

To further confirm that SUN treatment protocol used in this study is associated with cardiotoxicity, the effects of SUN on serum cardiotoxicity enzymatic indices, CK-MB and LDH, were studied in normal and carnitine-supplemented rats (Fig. 5). On the other hand, daily administration of SUN (25 mg/kg) for 28 successive days resulted in a significant 89% and 95% increase in serum CK-MB (A) and LDH (B), respectively, as compared to the control group. On the contrast, carnitine supplementation (200 mg/kg/day) for 28 successive days showed non-significant changes. Interestingly, daily administration of l-carnitine concomitant with SUN resulted in a complete reversal of SUN-induced increase in serum CK-MB and LDH to the control values.

**Fig. 6:**
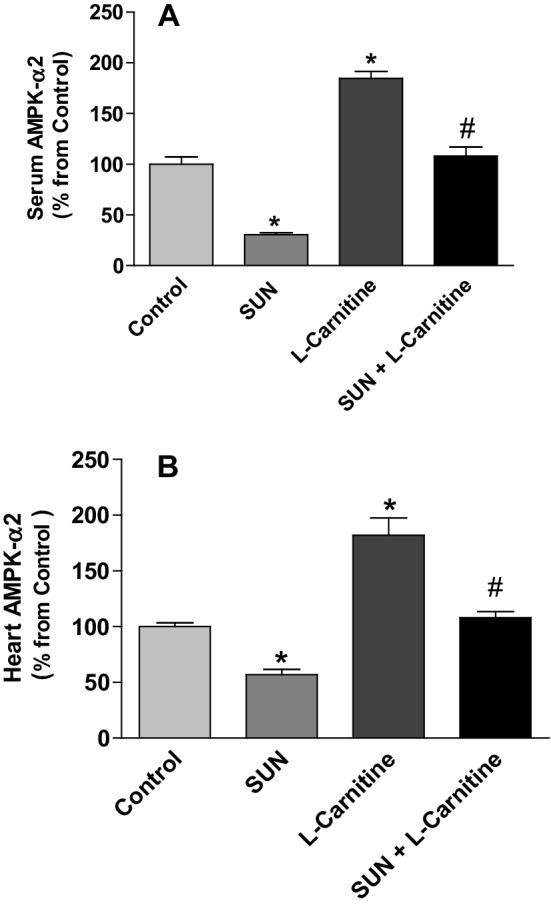
Effects of Sunitinib, l-carnitine, and their combination on the level of AMPK-α2 in serum (**a**) and rat heart tissues (**b**). Data are presented as mean ± S.E.M. (*n* = 10). Asterisk and ash symbols indicate significant change from control and SUN, respectively, at *p* < 0.05 using ANOVA followed by Tukey–Kramer as a post ANOVA test

Figure 6 shows the effect of SUN, l-carnitine, and their combination on the level of AMPK-α2 in serum (A) and rat heart tissues (B). Treatment with SUN (25 mg/kg/day) for 28 successive days resulted in a significant 70% and 43% decrease in AMPK-α2 level in serum and cardiac tissues, respectively, as compared to the control group. In contrast, daily administration of l-carnitine (200 mg/kg/day) for 28 successive days resulted in a significant 84% and 82% increase in AMPK-α2 level in serum and cardiac tissues, respectively, as compared to the control group. Administration of l-carnitine concomitant with SUN for 28 successive days resulted in a complete reversal of SUN-induced decrease in AMPK-α2 level in serum and rat heart tissues to the control values.

**Fig. 7:**
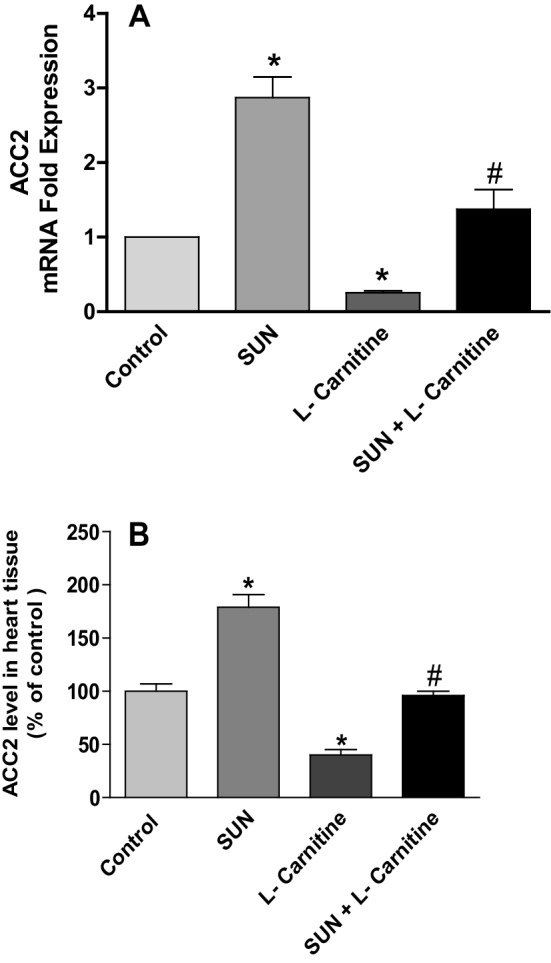
Effects of Sunitinib, l-carnitine, and their combination on mRNA expression (**a**) and protein level (**b**) of ACC2 in rat heart tissues. Data are presented as mean ± S.E.M. (*n* = 10). Asterisk and ash symbols indicate significant change from control and SUN, respectively, at *p* < 0.05 using ANOVA followed by Tukey–Kramer as a post ANOVA test

Acetyl-CoA carboxylase 2 (ACC2) is an enzyme expressed in the heart tissues and responsible for the carboxylation of acetyl-CoA to malonyl-CoA. Figure 7 shows the effects of SUN, l-carnitine, and their combination on ACC2 mRNA expression (A) and protein level (B) in rat heart tissues. Treatment with SUN (25 mg/kg/day) for 28 successive days resulted in a significant 187% and 79% increase in mRNA expression and protein level of ACC2 in rat heart tissues, respectively, as compared to the control group. On the other hand treatment with l-carnitine (200 mg/kg/day) for 28 successive days significantly decreased ACC2 mRNA expression and protein level in heart tissues by 75% and 60%, respectively, as compared to the control group. Intriguingly, daily administration of l-carnitine concomitant with SUN resulted in a complete reversal of SUN-induced increase in ACC2 mRNA expression and protein level in rat heart tissues to the control values.

**Fig. 8:**
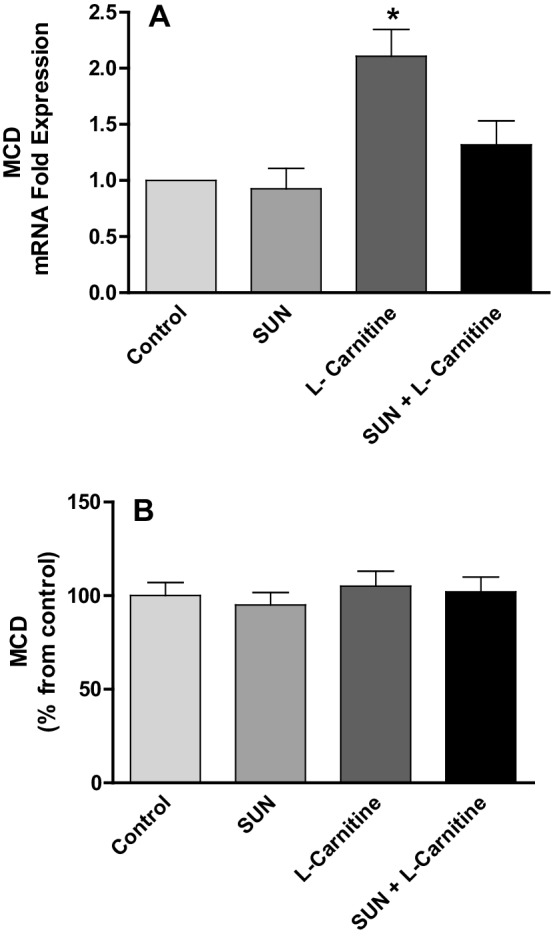
Effects of Sunitinib, L-carnitine, and their combination on mRNA expression (**a**) and protein level (**b**) of MCD in rat heart tissues. Data are presented as mean ± S.E.M. (*n* = 10). Asterisk indicate significant change from control at *p* < 0.05 using ANOVA followed by Tukey–Kramer as a post ANOVA test

Malonyl-CoA decarboxylase (MCD) is an enzyme expressed in the heart and catalyzes the decarboxylation of malony-CoA to acetyl-CoA. The effects of SUN, l-carnitine and their combination on MCD mRNA expression and protein level in rat heart tissues are shown in Fig. 8. Treatment with SUN (25 mg/kg/day) for 28 successive days resulted in non-significant change in MCD mRNA expression (A) and protein level (B) in rat heart tissues as compared to the control group. On the other hand, treatment with l-carnitine (200 mg/kg/day) for 28 successive days showed non-significant change in ACC2 protein level, but significantly increased mRNA expression by 101% in rat heart tissues as compared to the control.

**Fig. 9:**
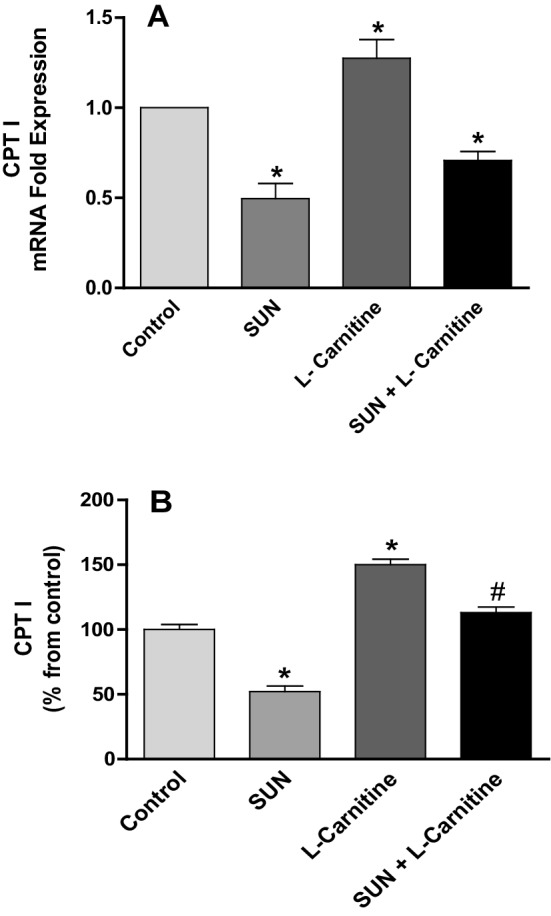
Effects of Sunitinib, l-carnitine, and their combination on CPT I mRNA expression (**a**) and protein level (**b**) in rat heart tissues. Data are presented as mean ± S.E.M. (*n* = 10). Asterisk and ash symbols indicate significant change from control and SUN, respectively, at *p* < 0.05 using ANOVA followed by Tukey–Kramer as a post ANOVA test

To investigate the effect of SUN on mitochondrial transport of LCFA, mRNA expression and protein level of CPT I were measured in rat heart tissues (Fig. 9). Daily administration of SUN (25 mg/kg) for 28 days resulted in a significant 50% and 48% decrease in CPTI mRNA expression (A) and protein level (B) in rat heart tissues, respectively, as compared to the control group. On contrast, carnitine supplementation for 28 successive days significantly increased mRNA expression and protein level in heart tissues by 28% and 50%, respectively, as compared to the control group. Interestingly, daily administration of l-carnitine concomitant with SUN completely reversed SUN-induced decrease in CPTI protein level but not mRNA expression to the control values.

**Fig. 10:**
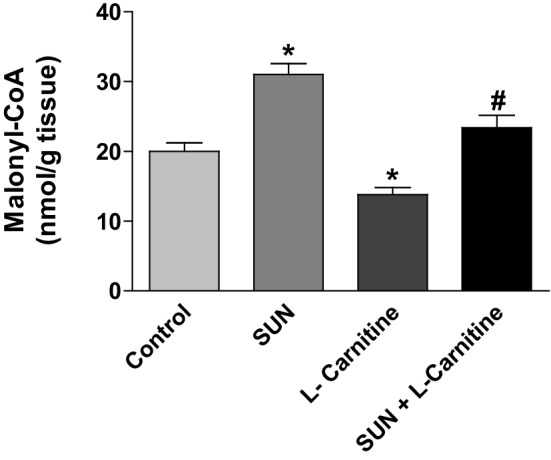
Effects of Sunitinib, l-carnitine, and their combination on the level of malonyl-CoA in rat heart tissue. Data are presented as mean ± S.E.M. (*n* = 10). Asterisk and ash symbols indicate significant change from control and SUN, respectively, at *p* < 0.05 using ANOVA followed by Tukey–Kramer as a post ANOVA test

To study the effects of SUN, l-carnitine, and their combination on the activity of CPT I in cardiac tissues, the level of malonyl-CoA, the well-known physiological and potent inhibitor of CPT I enzyme, was measured using HPLC (Fig. 10). Daily administration of SUN (25 mg/kg) for 28 days resulted in a significant 55% increase in malonyl-CoA level in rat heart tissues, as compared to the control group. On the other hand, daily administration of l-carnitine alone for 28 successive days significantly decreased the level of malonyl-CoA in heart tissues by 30% as compared to the control group. Interestingly, daily administration of l-carnitine concomitant with SUN completely reversed SUN-induced increase in cardiac malonyl-CoA level to the control values.

**Fig. 11:**
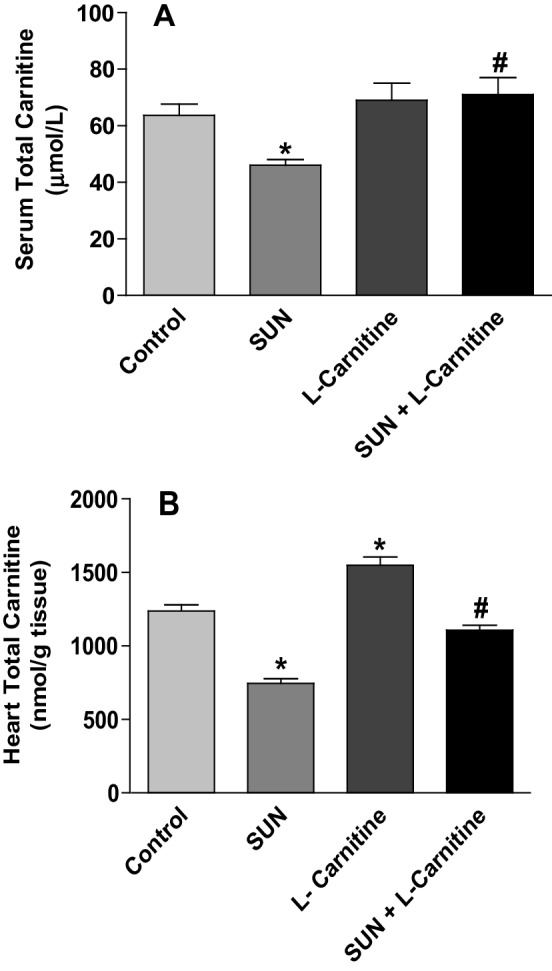
Effects of Sunitinib, l-carnitine, and their combination on total carnitine levels in serum (**a**) and rat heart tissues (**b**). Data are presented as mean ± S.E.M. (*n* = 10). Asterisk and ash symbols indicate significant change from control and SUN, respectively, at *p* < 0.05 using ANOVA followed by Tukey–Kramer as a post ANOVA test

Figure 11 shows the effects of SUN, l-carnitine and their combination on total carnitine levels in serum (A) and rat heart tissues (B). Administration of SUN (25 mg /kg/day) for 28 successive days resulted in a significant 27% and 40% decrease in total carnitine level in serum and cardiac tissues, respectively, as compared to the control group. On the other hand, Daily administration of l-carnitine (200 mg/kg/day) for 28 days resulted in a significant 25% increase in total carnitine level in cardiac tissues and non-significant change in serum carnitine level, as compared to control group. Remarkably, daily administration of l-carnitine concomitant with SUN resulted in a complete reversal of SUN-induced decrease in total carnitine level in serum and rat hear tissues to the control values.

**Fig. 12:**
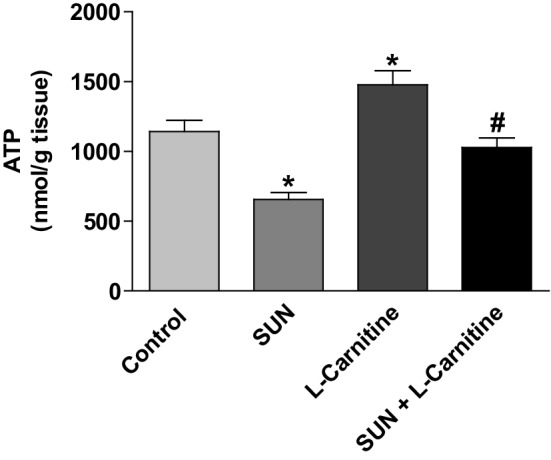
Effects of Sunitinib, l-carnitine, and their combination on the level of ATP in rat heart tissue. Data are presented as mean ± S.E.M. (*n* = 10). Asterisk and ash symbols indicate significant change from control and SUN, respectively, at *p* < 0.05 using ANOVA followed by Tukey–Kramer as a post ANOVA test

Figure 12 shows the effect of SUN, l-carnitine, and their combination on the level of ATP in rat cardiac tissues. Daily administration of SUN (25 mg/kg) for 28 successive days resulted in a significant 43% decrease in ATP content in rat heart tissues as compared to control group. On the other hand, treatment with l-carnitine (200 mg/kg/day) for 28 successive days resulted in a significant 30% increase in ATP level as compared to control group. Interestingly, daily administration of l-carnitine concomitant with SUN completely reversed SUN-induced decrease in ATP level in rat heart tissues to the control values.

## Discussion

In the current study, SUN treatment protocol used to develop cardiotoxicity is confirmed by increasing heart weight, cardiac index and cardiotoxicity enzymatic indices, LDH and CK-MB, as well as severe histopathological changes which are manifested as scattered chronic inflammatory cells with focal fragmentation of myocardial fibers and loss of nuclei. Our results are consistent with previous experimental studies which have reported that SUN therapy was associated with cardiac hypertrophy, myocardial fibrosis, and increased cardiac enzyme [9, 10, 13]. Recently, Blanca et al. reported that SUN-induced myocardial fibrosis and inflammation was as a consequence of oxidative stress and is involved in SUN-induced cardiotoxicity [13]. SUN-induced oxidative stress and lipid peroxidation of cardiac membranes might signify the myocardial cell damage with the consequent leakage of these markers from damaged cardiac myocytes. Interestingly, daily administration of l-carnitine (200 mg/kg) for 28 days completely reversed SUN-induced increase in cardiac enzymes, cardiac index and myocardial fibrotic process to the normal values. It is worth mentioning that histological damage-induced by SUN in cardiac tissues and the protection achieved by carnitine supplementation are similar to those observed by Blanca et al. [13] although exposure time to both SUN and carnitine is different.

Under similar experimental condition, Blanca et al. confirmed that daily administration of l-carnitine (400 mg/kg) in drinking water offered complete protection against SUN-induced cardiotoxicity via down-regulating the myocardial expression of inflammatory and fibrotic markers, oxidative stress markers and NF-kB [13]. In this regard, carnitine treatment resulted in a down-regulation of nitrotyrosine, NOX2, IL-1b, IL-6, TGF-b1, collagen type I and tissue inhibitor of metalloproteinase-1, together with an upregulation of IL-10 and matrix metalloproteinase-9 [13]. Also, antioxidant, antiinflammatory and anti-fibrotic effects of l-carnitine have been previously reported in earlier studies [13, 31, 41].

Adenosine Monophosphate-Activated Protein Kinase is an important regulator of LCFA oxidation and glucose uptake in the heart [19]. Data presented in the current study showed that SUN decreased AMPK-α2 levels in serum and cardiac tissues. The role of AMPK and its inhibition in SUN-induced cardiotoxicity has been reported in several in vivo and in vitro models of SUN-induced cardiotoxicity [14–16]. Acetyl-CoA carboxylase (ACC) is the AMPK substrate that generates malonyl-CoA from the carboxylation of cytosolic acetyl-CoA. It is well documented that phosphorylation of ACC2 by AMPK is associated with inactivation of ACC2 with the consequent increase of mitochondrial transport and oxidation of LCFA [21]. Our results showed that SUN significantly increased ACC2 mRNA expression and protein level. This effect could be secondary event following SUN-induced inhibition of AMPK with the consequent decrease in the phosphorylation of its substrate, ACC2. If this hypothesis is correct, phosphorylation of ACC2 (P-ACC2) should decrease following treatment with SUN. In fact, earlier study reported that SUN significantly decreased P-ACC2 protein expression secondary to the inhibition of AMPK in mice treated with SUN and in cardiac myocytes in culture [17]. Furthermore, SUN significantly reduced activity of AMPK following treatment with the AMPK activator, AICAR, as evidenced by decreased P-ACC2 protein expression and the degree of inhibition was parallel to that seen with the AMPK inhibitor, compound C [17]. It is worth mentioning that results of ACC2 and AMPK presented in our study warrants monitoring the activity and the expression of phosphorylated proteins codified by these genes. Our results showed that SUN decreased AMPK with the consequent inhibition of ACC2 phosphorylation and this could increase in ACC2 activity resulting in enhanced production of malonyl-CoA in cardiac tissues from acetyl-CoA. Consistent with this hypothesis, data presented in the current study demonstrated that SUN increased malonyl-CoA level in cardiac tissues. Since our results showed that SUN induced no effect on MCD mRNA expression and protein level, therefore increased carboxylation of acetyl-CoA by ACC2 but not degradation of malonyl-CoA by MCD is the only explanation for the observed increase in malonyl-CoA production in cardiac tissues. It is well known that malonyl-CoA is the key regulator of LCFA oxidation in the heart because it is the physiological and potent inhibitor of CPT I which transports LCFA from cytoplasm into mitochondria [42–45]. Our results showed that SUN significantly decreased mRNA and protein expression of CPT I. Since CPT I is the major player in mitochondrial transport and oxidation of LCFA, its inhibition by SUN could inhibit the translocation of LCFA from cytoplasmic compartment into mitochondrial compartment where β-oxidation enzymes are located. Several experimental studies have confirmed that the inhibition of CPT I plays an important role in cancer chemotherapy-dependent [46–48] and independent [49–51] cardiomyopathies.

Data presented in the current study have demonstrated that SUN significantly decreased total carnitine in cardiac tissues. It seems that our results are unique since there is no available experimental or clinical data regarding the role of endogenous carnitine during the development of SUN-induced cardiotoxicity. Decreased myocardial carnitine content and its contribution in cancer chemotherapy-induced cardiotoxicity have been well documented [30]. Under our experimental condition, carnitine supplementation increased myocardial carnitine content and completely restored SUN-induced decrease in carnitine in both serum and heart tissues to its normal values. Moreover, carnitine supplementation for 28 successive days significantly increased mRNA and protein expression of CPT I which are consistent with earlier studies [46]. This observed increase in CPT I expression by carnitine supplementation in this study could be secondary event following l-carnitine-induced decrease in malonyl-CoA production, the well-known potent inhibitor of CPT I. Accordingly, it could be suggested that carnitine supplementation for 28 days will enhance mitochondrial transport of LCFA through CPT I with the consequent enhancement of β-oxidation and ATP production and this could be the underlying protective mechanism of l-carnitine against SUN-induced cardiotoxicity. Consistent with this hypothesis is that carnitine supplementation for 28 days increased AMPK in serum and cardiac tissues. Using in vitro renal tubular cells and in vivo mice models of carboplatin-induced renal injury, Sue et al. reported that l-carnitine prevented carboplatin-mediated apoptosis through activation of AMPK [52]. Using rat L6 muscle cells, Zhang et al., investigated how TNF-α preferentially impaired insulin downstream signaling and what the role acetyl-l-carnitine played to alleviate this insulin resistance state [53]. The authors found that acetyl-l-carnitine inhibited TNF-α-induced insulin resistance via activating AMPK signaling, thus increasing rates of skeletal muscle fatty-acid oxidation, leading to reduced malonyl-CoA and increased long chain fatty acyl-CoA flux into the mitochondria [53]. In the current study, the observed increase in mRNA expression of MCD and normal level of MCD protein in the presence of carnitine could be due to post-transcriptional modifications. Conversely, carnitine supplementation decreased expression of ACC2 mRNA and protein level. Accordingly, one can anticipate that the observed decrease in malonyl-CoA level in cardiac tissues by carnitine supplementation could be secondary to decreasing its synthesis from its ultimate precursor, acetyl-CoA, by ACC2 rather than its degradation by MCD. Results from this study demonstrated that carnitine supplementation completely reversed SUN-induced decrease of CPT I and increase of ACC2 to the normal values. In conclusions, data obtained from the current study suggest that (1) SUN inhibits AMPK downstream signaling via the inhibition of the expression CPT I with the consequent inhibition of mitochondrial transport and oxidation of LCFA in cardiac tissues. (2) SUN therapy decreased myocardial carnitine content with the consequent carnitine deficiency. (3) The observed decrease in carnitine and CPT I expression in cardiac tissues by SUN was parallel to the increase in cardiac index, cardiac enzymes and myocardial fibrosis which may point to the possible contribution of carnitine deficiency and the inhibition of CPT I as possible mechanisms in SUN-induced cardiotoxicity. (4) Carnitine supplementation attenuates SUN-induced cardiotoxicity via increasing myocardial carnitine content and modulating key genes engaged in LCFA oxidation.
